# Node Position Determines Sweetpotato (*Ipomoea batatas* [L.] Lam.) Cutting Performance: Toward a Targeted Inverted Planting Strategy

**DOI:** 10.3390/plants15172548

**Published:** 2026-08-22

**Authors:** Alfredo Morales, Dania Rodríguez, Peiyong Ma, Zhaodong Jia, Qian Zhang, Shuai Liu, Xiaofeng Bian, Iván Javier Pastrana Vargas, Yinhua Chen, Michel Leiva-Mora, Orelvis Portal, Luis Rodrigo Saa

**Affiliations:** 1 Research Institute of Tropical Roots and Tuber Crops (INIVIT), Santo Domingo 53000, Cuba; daniarodriguezdelsol@gmail.com; 2 Institute of Food Crops, Jiangsu Academy of Agricultural Sciences (JAAS), Nanjing 210095, China; andympy@163.com (P.M.); jzdgood162@126.com (Z.J.); zhangqian508@163.com (Q.Z.); 18761698534@163.com (S.L.); bianxiaofeng2@163.com (X.B.); 3 Department of Agronomic Engineering and Rural Development, Faculty of Agricultural Sciences, Universidad de Córdoba, Carrera 6 No. 77-305, Montería 230002, Colombia; ivanpastranav@correo.unicordoba.edu.co; 4 College of Tropical Crops, Hainan University, Haikou 570228, China; yhchen@hainanu.edu.cn; 5 Faculty of Agricultural Sciences, Technical University of Ambato, Cevallos 1801334, Ecuador; m.leiva@uta.edu.ec; 6 Department of Biology, Faculty of Agricultural Sciences, Central University "Marta Abreu" of Las Villas, Santa Clara 54830, Cuba; orelvispv@uclv.cu; 7 Agricultural Research Center, Faculty of Agricultural Sciences, Central University "Marta Abreu" of Las Villas, Santa Clara 54830, Cuba; 8 Department of Biological and Agricultural Sciences, Faculty of Exact and Natural Sciences, Private Technical University of Loja (UTPL), Loja 1101608, Ecuador

**Keywords:** nodes, rhizogenesis, rooting of cuttings, tuberization, vegetative propagation

## Abstract

Sweetpotato (*Ipomoea batatas* [L.] Lam.) is a vital crop for food security, yet yields in Cuba remain below potential (2.5–10.0 t ha^−1^) due to suboptimal propagation practices. This study investigated the impact of nodal position (1–22) on root development in stem cuttings of cv. ‘INIVIT B-240’ under controlled conditions. Root primordia (24 h), fibrous roots (7 days), and storage roots (60 days) were evaluated. Intermediate nodes (8–12) exhibited superior rhizogenic capacity, with node 10 showing the highest root primordia density (3.0 ± 3) and nodes 10–12 producing the most fibrous roots (24.6–26.7 roots/cutting). Storage root formation was optimal at nodes 5–13, particularly nodes 8–11 (1.30+ roots/node; highest biomass). Multivariate analysis (Integrated Performance Index) confirmed nodes 8–12 as the most productive cluster. These findings challenge traditional apical-cutting practices, showing that mid-stem nodes optimize both rooting and tuberization. We propose inverted planting of pre-apical sections to bury high-performance nodes, potentially increasing yields in resource-limited systems.

## 1. Introduction

Sweetpotato (*Ipomoea batatas* [L.] Lam.) ranks among the world’s most important food crops, holding 12th place in global production with 93.5 million tons annually [1]. Its nutritional value, short cycle, and drought tolerance make it key for food security in tropical regions [2,3]. Despite this, Cuban yields remain historically low (2.5–10.0 t ha^−1^) due to soil degradation, limited irrigation, pests, and clonal degeneration from continuous vegetative propagation [3,4]. 

A critical but less-studied factor is planting material quality. Over 90% of Cuban farmers use whole cuttings without nodal selection, ignoring physiological differences between stem segments [3]. Anatomical studies showed that adventitious roots emerge from stem nodes, with only a subset becoming storage roots, a process influenced by genetic and environmental factors [5,6,7,8,9,10]. However, these studies focused on early rooting stages and did not evaluate all stem nodes or quantify actual storage root formation. 

While Ma et al. [6] demonstrated that nodes 9–13 exhibit higher root primordia density and adventitious root development compared to nodes 3–8, it remains unclear how these anatomical differences translate into actual storage root yield, whether variations exist beyond node 13, and whether the optimal nodal position can be translated into a practical planting technique. 

Our study addresses these questions with a novel, applied approach. Unlike previous work, we: (1) evaluated all stem nodes (1–22) to identify unreported variations; (2) monitored root development up to 60 days after planting, covering the critical storage root formation phase; (3) quantified actual yield (storage root number and weight), not just initial rooting capacity; and (4) proposeed an inverted planting technique that buries high-performance nodes. This integrated approach uncovers node position–productivity relationships undetectable in prior studies and provides a practical solution to improve sweetpotato vegetative propagation in resource-limited systems. 

## 2. Results

**Figure 1:**
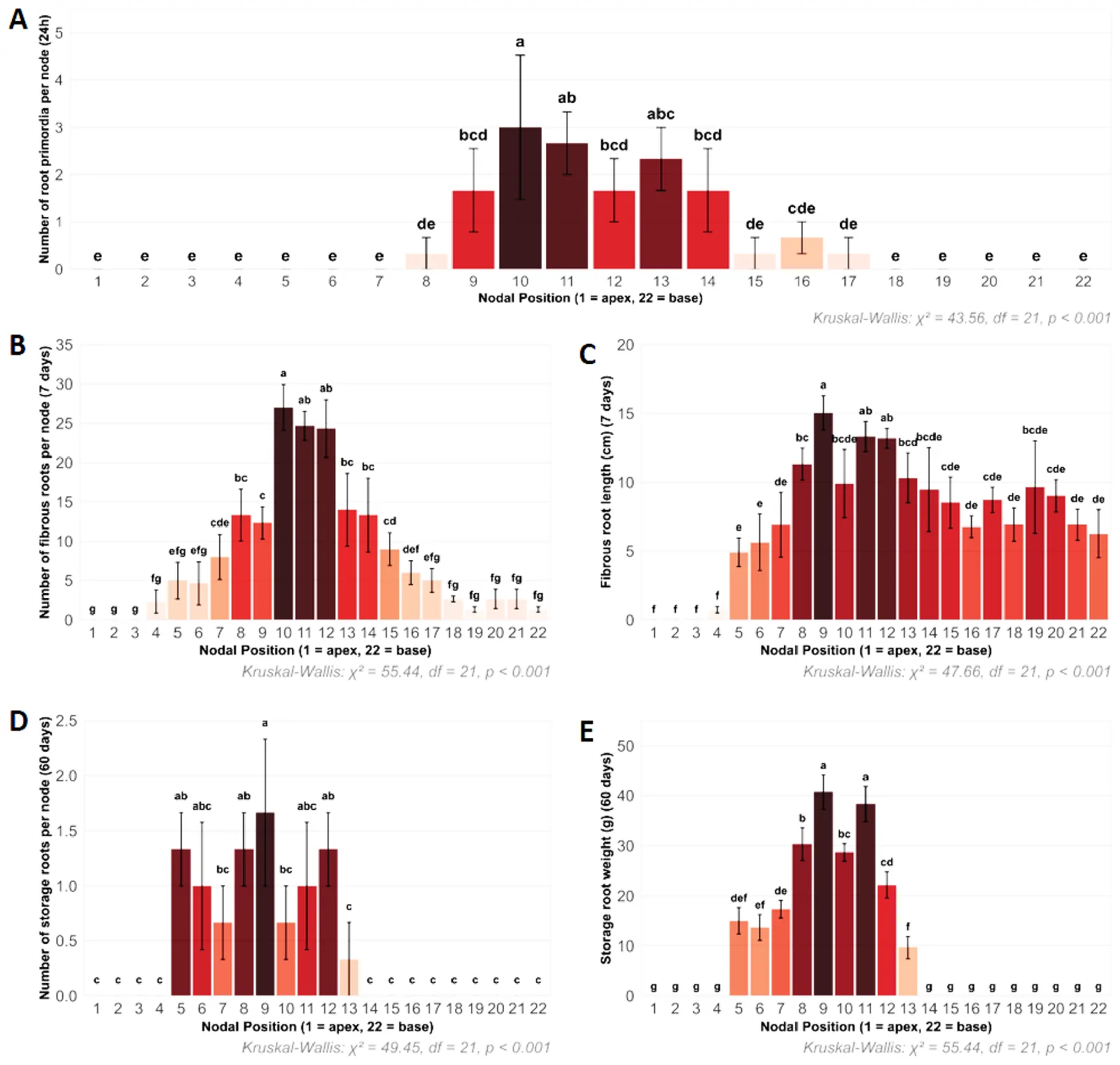
Root development parameters across sweetpotato nodal positions (nodes 1–22). (**A**) Root primordia number at 24 h; (**B**) fibrous root number at 7 days; (**C**) fibrous root length at 7 days; (**D**) storage root number at 60 days; (**E**) storage root weight at 60 days. Different letters indicate significant differences (*p* < 0.001, Kruskal–Wallis test with Dunn’s post hoc test). Values are means ± SE.

Statistical analysis revealed significant differences in root primordia formation at 24 h among the distinct nodes (χ^2^ = 43.56, *p* < 0.001). Node 10 showed the highest average number of root primordia (3.0 ± 1.5), while nodes 9, 11, 12, 13, and 14 formed an intermediate rooting capacity group, and nodes 1–8 and 15–22 showed the lowest performance (Figure 1A). This pattern indicates that nodes located in the middle stem region are the most suitable for early root initiation. 

At 7 days, nodes 10, 11, and 12 showed the highest production of fibrous roots (26.7 ± 4.5, 24.8 ± 2.2, and 24.6 ± 4.1 roots, respectively), while nodes 1–3 developed no roots. Nodes 8, 9, 13, and 14 formed a group with comparable performance, whereas apical nodes (4–6) and distal basal nodes (17–22) showed averages below five fibrous roots (Figure 1B). Fibrous root length was greatest at intermediate nodes (8–13), exceeding 10 cm, while young nodes (1–3) did not survive and basal nodes (17–22) showed reduced lengths compared to intermediate nodes (Figure 1C).

**Figure 2:**
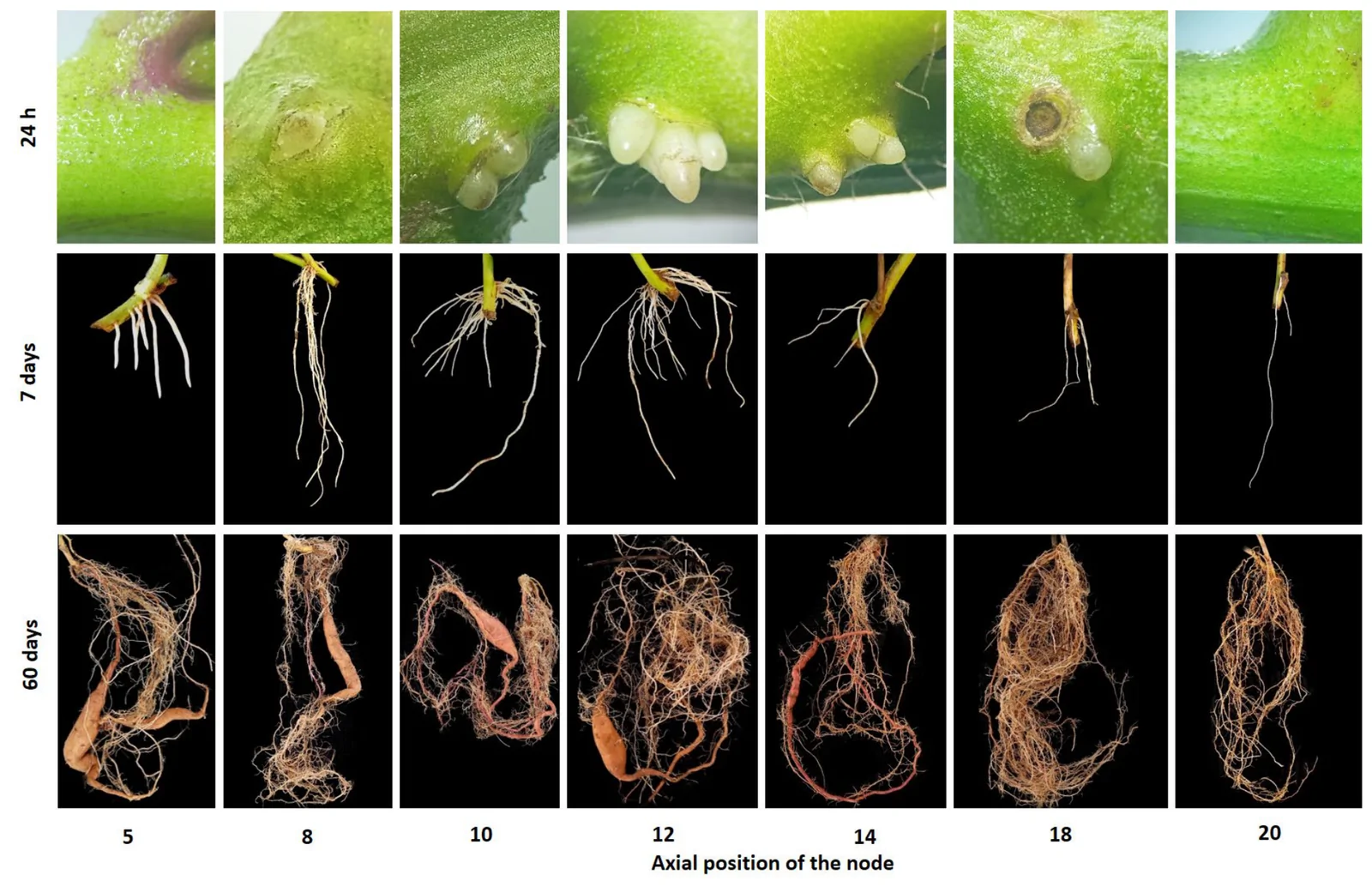
Visual representation of sweetpotato nodal development along the stem axial gradient at three key time points. Columns represent selected nodes (5, 8, 10, 12, 14, 18, and 20). Rows show: (**top**) root primordia at 24 h; (**middle**) fibrous root development at 7 days; (**bottom**) storage root formation at 60 days. This figure illustrates the superior rhizogenic capacity of intermediate nodes (8–12) compared to apical (5) and basal (14–20) nodes across all developmental stages.

At 60 days, nodes 5–13 demonstrated the capacity to form storage roots, with nodes 5, 8, 9, and 12 showing the highest number (>1.30 storage roots per node). Nodes 14–22 did not form storage roots. Notably, nodes 18–22 showed poor survival by harvest time. Storage root weight was exceptionally high at nodes 8–11, with significant differences (*p* < 0.001) compared to the rest of the nodes (Figure 1D,E). These results reveal a clear physiological gradient in which nodes 8–12 constitute the optimal zone for storage root formation, while apical and basal nodes show limited performance (Figure 2). 

**Figure 3:**
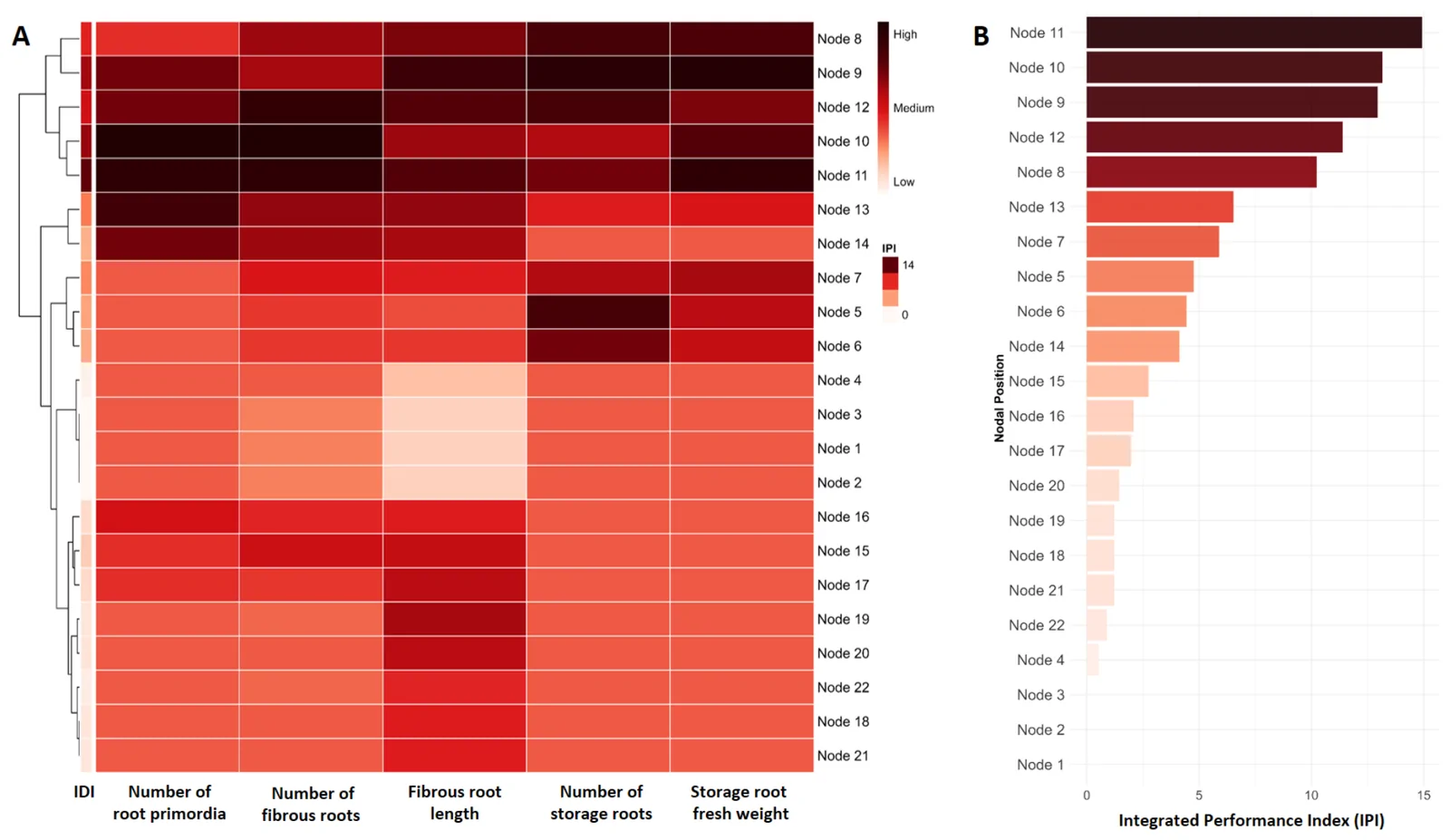
Multivariate analysis of root performance in nodal sweetpotato cuttings. (**A**) Heatmap with hierarchical clustering of nodes, variables (root primordia, fibrous roots, and storage roots), and Integrated Performance Index (IPI) annotation; (**B**) node ranking by IPI (higher values indicate better performance).

Multivariate analyses revealed clear variability in root performance based on nodal position in sweetpotato cuttings. The heatmap showed that nodes 8–12 formed a distinct cluster associated with higher values of root primordia (24 h), fibrous root number and length (7 days), and storage root weight (60 days). This pattern correlated with the highest scores in the Integrated Performance Index (IPI), confirming that middle stem nodes (8–12) are optimal for propagation (Figure 3A). 

IPI annotation on the heatmap demonstrated that basal (1–4) and senescent nodes (15–22) exhibited inferior performance, coinciding with the absence of storage roots and low primordia counts. The node ranking by IPI clearly showed that nodes 8–12 obtained the highest scores, while apical and basal extremes obtained the lowest (Figure 3B). These results suggest that tissue physiological age, ranging from young apical meristems to fully lignified basal sections, is a critical determinant of rooting capacity. 

## 3. Discussion

Our results demonstrate that intermediate nodes (8–12) exhibit the highest capacity for both fibrous and storage root formation, while apical (1–4) and basal nodes (17–22) show significantly poorer performance. The poor survival of basal nodes (18–22) is consistent with their senescent state and advanced lignification of the stem tissue. These older sections lack functional leaves, which are essential for providing photoassimilates and hormonal signals required for root initiation and establishment. This pattern can be attributed to hormonal gradients along the stem (auxins, cytokinins, and ethylene) that regulate the initiation and development of adventitious roots. Ma et al. [6] demonstrated that intermediate nodes possess a higher density of preformed root primordia, facilitating rapid root emergence. In addition, Belehu [11] described these primordia as latent structures that activate in response to hormonal and environmental signals. Intermediate nodes represent an optimal stage of cellular development, with sufficient maturity to respond to differentiation signals without losing physiological plasticity, unlike apical nodes, which lack maturity, and basal nodes, which enter senescence [5].

Beyond nodal position, the physiological state of leaves is a key determinant. Nodes with functional leaves (5–18) outperformed leafless nodes (1–4 and 19–22), since leaves at intermediate nodes operate at maximum photosynthetic capacity and modulate the synthesis and transport of hormones. This flow of photoassimilates provides energy for fibrous root elongation and substrate for storage root thickening. 

Our findings partially align with Ma et al. [6] but extend their results by demonstrating that the initial rooting advantage does not always translate into greater storage root formation, since younger nodes (5–8) also developed storage roots, although with lower weight. This suggests that the potential of primordia to become storage roots depends on later physiological conditions, such as carbohydrate availability and hormonal regulation [11]. Netsai et al. [5] reported that intermediate cuttings generate thicker and heavier roots; our results support this and reveal that specific nodal positions (nodes 10 and 12) show the optimal balance between initial rooting and biomass accumulation. 

Recent studies have highlighted the importance of nodal management in sweetpotato. Song et al. [12] reported that increasing node number to 8 or 10 per cutting improved storage root yield by 23–30% and that flat planting increased yield by 18–20% compared to slanted planting. Ossai et al. [13] also showed that nodal explant type and substrate significantly influenced vine production. However, neither study evaluated nodal position across the full stem gradient (1–22) or proposed a targeted strategy based on nodal performance. 

**Figure 4:**
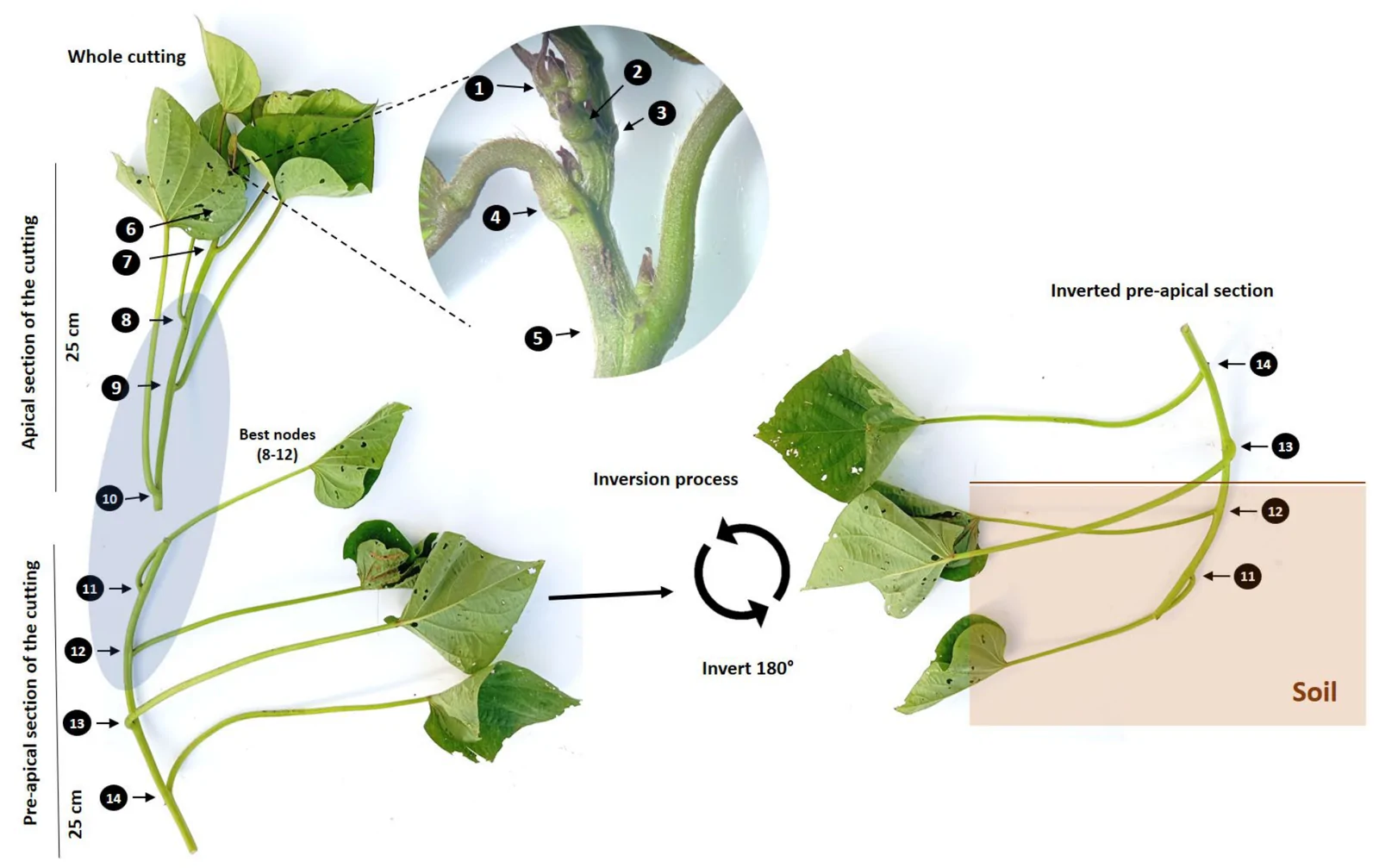
Inverted planting proposal: nodes 11–12 are buried, ensuring contact with soil moisture and nutrients and maximizing storage root formation.

A critical practical implication arises from our findings. Traditionally, apical cuttings of ~25 cm (nodes 1–10) are recommended, assuming that their physiological youth ensures better rooting. However, our results demonstrate that the optimal nodes for storage roots (8–12) do not always coincide with the apical section. In cultivars with long internodes, nodes 11–12 (among the most productive) fall outside the standard apical cutting and remain above ground. In many countries, seed scarcity forces the use of pre-apical sections (the four nodes following the apical one), but in practice they are not buried, leaving nodes 11 and 12 outside the soil. To solve this, we propose inverted planting of the pre-apical section. By inverting this section, nodes 11 and 12 are buried, ensuring their contact with soil moisture and nutrients and maximizing storage root formation. This technique has the potential to transform an underutilized resource into productive planting material, offering a practical, low-cost strategy that may improve sweetpotato propagation in resource-limited systems (Figure 4). 

## 4. Materials and Methods

### 4.1. Plant Material and Experimental Setup

Sweetpotato cuttings of the commercial cultivar ‘INIVIT B-240’ were obtained from healthy mother plants grown under greenhouse conditions. Mother plants were propagated through tissue culture and maintained under greenhouse conditions for 3 months prior to cutting collection. A total of 10 healthy mother plants of the commercial cultivar ‘INIVIT B-240’ were used. Selection criteria for ‘healthy’ plants included: (1) absence of visible pest or disease symptoms, (2) vigorous growth with uniform stem diameter (6–8 mm), and (3) fully expanded green leaves without signs of chlorosis or necrosis. 

Stems were segmented into individual nodes (1 to 22), numbered from the apex (node 1 = apical meristem) to the base (node 22 = senescent zone). Each node contained an axillary bud and approximately 2 cm of adjacent tissue above and below the leaf insertion point. Each node was trimmed to a standard length of 2 cm above and 2 cm below the leaf insertion point, using a sharp scalpel to ensure consistency and minimize tissue damage. Nodes with visible damage, abnormal morphology, or non-uniform size were discarded. Key morphological distinctions were observed: nodes 1–4 had closed leaf buds; nodes 5–18 had fully expanded leaves; and nodes 19–22 were leafless due to natural senescence.

### 4.2. Growing Conditions

Black polyethylene bags (15 cm × 20 cm, 2 kg capacity) with drainage holes were filled with sterilized (121 °C for 20 min) peat:vermiculite substrate (3:1 *v*/*v*). On 15 January 2025, nodes were planted at a 2 cm depth with the bud oriented upward. Initial watering to field capacity was maintained through capillary action. Greenhouse conditions were maintained at 25 ± 2 °C, 70–80% relative humidity, and a natural photoperiod. The experiment was conducted twice independently (January–March and April–June 2025) under identical conditions, and the data presented are the average of both runs. 

### 4.3. Experimental Design

A completely randomized design was employed with 10 replicates per node per evaluation time (24 h, 7 days, and 60 days), totaling 30 replicates per node and 660 experimental units overall. Destructive sampling was used for each evaluation, ensuring that root measurements were taken without disturbing the remaining experimental units. Root primordia were counted at 24 h using stereomicroscopy (10×), considering emergences ≥1 mm from the node base. At 7 days, fibrous root number and length were recorded. At 60 days, storage root number and weight were determined. All variables were measured following standard protocols. 

### 4.4. Statistical Analysis 

All variables violated normality (Shapiro–Wilk, *p* < 0.001) and homoscedasticity (Levene’s, *p* < 0.001) assumptions, justifying non-parametric methods. The Kruskal–Wallis test was used to determine significant differences among nodes, with Dunn’s post hoc test and Bonferroni correction (α = 0.05). Results are presented with significance letters (a, b, c…). For integrated visualization, a heatmap with hierarchical clustering (Euclidean distance and complete linkage) was constructed using standardized values (z-scores) of five key variables. The heatmap included the IPI: 0.3 × (primordia) + 0.2 × (fibrous root number) + 0.1 × (fibrous root length) + 0.2 × (storage root number) + 0.2 × (storage root weight). Nodal ranking was generated by plotting IPI scores (mean ± SE) in descending order. All analyses were performed in R v4.3.1 using pheatmap (v1.0.12) and ggplot2 (v3.4.2). 

## 5. Conclusions

This study demonstrates that nodal position significantly influences root development and storage root formation in sweetpotato cuttings, with intermediate nodes (8–12) exhibiting the greatest rhizogenic capacity and storage root biomass accumulation, while apical (1–4) and basal nodes (17–22) show limited or null development. These findings challenge the traditional practice of using whole cuttings without nodal selection, since the most productive nodes (11–12) in the pre-apical section often remain exposed above ground when using standard 25 cm cuttings, particularly in cultivars with long internodes. To address this, we propose inverted planting of the pre-apical section, a simple, low-cost technique that ensures the burial of productive nodes, thereby maximizing storage root formation. This practical approach offers a tangible strategy to improve sweetpotato propagation efficiency in resource-limited systems, transforming previously underutilized planting material into a productive resource with the potential to increase yields without additional inputs, although further field trials are needed to validate these benefits across diverse environments and genotypes. 

## Data Availability

The raw data supporting the conclusions of this article will be made available by the authors on request.
